# Controlling the Digital to Analog and Multilevel Switching in Memristors Based on Zr-Doped HfO_2_ by Interface Engineering

**DOI:** 10.3390/ma18184352

**Published:** 2025-09-17

**Authors:** Cong Han, Haiming Qin, Weijing Shao, Hanbing Fang, Hao Zhang, Xinpeng Wang, Yu Wang, Yi Liu, Yi Tong

**Affiliations:** 1 College of Integrated Circuit Science and Engineering, Nanjing University of Posts and Telecommunications, No. 9 Wenyuan Road, Nanjing 210023, China; 2 Suzhou Laboratory, 388 Roushui Road, Suzhou 215123, China; 3 Gusu Laboratory of Materials Science, No. 388 Ruoshui Road, Suzhou 215123, China

**Keywords:** oxide, memristors, binary switching, digital-to-analog, high density storage

## Abstract

Metal oxides are the most widely used material for the resistive switching layer of memristors. Nevertheless, the majority of oxide-based memristors exhibit binary switching, restricting the emulation of neuronal synaptic behaviors. In this paper, the shift from digital-to-analog switching behavior is achieved by inserting an Al_2_O_3_ layer atop Zr-doped HfO_2_. The TiN/Al_2_O_3_/HZO/W/Si device exhibits long resistance state retention time and consistency. In addition, by applying a varying voltage, the device exhibits up to 20 continuous resistance states, which is highly significant for high-density storage. Upon the application of a programmable pulse signal, the device’s conductance undergoes continual alteration, reflecting long-term potentiation (LTP) and long-term depression (LTD) synaptic characteristics. The conduction mechanism of the device is studied through physical model fitting and schematic diagrams.

## 1. Introduction

Brain-inspired neuromorphic computing is considered to be a computing architecture with great application potential, anticipated to address the memory wall issue associated with the conventional von Neumann architecture [1,2,3,4,5]. In hardware-implemented neuromorphic computing systems, the neural synapse is a critical component, serving to connect neurons and store the connection weights between them, hence occupying a significant portion of the system’s space [6,7]. Among several electronic synaptic devices, memristors, as two-terminal devices, show many advantages, including miniaturization, fast switching speed, and low power consumption, making them the most promising candidates for artificial synapses [8,9,10,11,12]. Most of memristors are based on metal oxide resistive switching layers, including titanium dioxide [13,14], silicon dioxide [15,16], and hafnium dioxide [17,18]. Among them, memristors based on hafnium dioxide exhibit significant Complementary Metal-Oxide-Semiconductor (CMOS) compatibility and have received widespread attention [19]. However, memristors based on hafnium dioxide typically show binary switching behavior [20,21], also called digital switching behavior. For ideal synaptic devices, linearity is a crucial parameter to improve the accuracy of neuromorphic computing. Therefore, efficient synaptic devices require good analog switching behavior rather than binary. Here, the analog-type behavior represents a gradual switching phenomenon, that is, there is no obvious resistance jump point during the resistive behavior [22,23]. The sudden change of resistance value during the switching process is called digital switching behavior, also known as binary-type switching behavior [24].

The switching behavior of oxide-based memristors is generally determined by the formation of oxygen vacancy pathways inside the resistive material. Under the applied voltage stimulus, the conductive filaments composed of oxygen vacancies usually form and break suddenly during the resistive switching process, resulting in digital switching behavior [25,26,27]. To address this problem, researchers have employed various approaches to improve the analog switching behavior of memristors. L. Chang et al. achieved the digital-to-analog switching behavior by inserting the thin suboxide interface layer in TiO_2_-based memristor devices [22]. A. Saleem et al. enhanced the linearity using TiW and TiWO_x_ layers in TiO_x_-based memristors [23]. L. Li et al. realized the digital-to-analog switching through modulating the oxide thickness in HfO_2_-based memristors [28]. From this view, incorporating an interface layer is an important way to change the switching behavior of memristors.

In this work, we selected Zr-doped hafnium dioxide (HZO) film as the resistive switching layer. HZO is recognized for its different storage properties, such as ferroelectric properties in the crystalline state and resistive switching behavior in the amorphous state. Therefore, the study of this material system is interesting. We proposed a TiN/Al_2_O_3_/HZO/W structure to improve the switching behavior of the device. As a result, compared with the HZO memristor, this device exhibits bipolar non-volatile analog switching behavior, capable of storing up to 20 resistance states, with each state persisting for over 10^3^ s. Testing various devices revealed that the high and low resistance states were consistently maintained, demonstrating excellent consistency. This holds substantial importance for neuromorphic computing and high-density storage.

## 2. Materials and Methods

The process flow is as follows: first, 50 nm of tungsten (W) metal used as the bottom electrode is DC magnetron sputtered onto the Si/SiO_2_ substrate, with a sputtering power of 350W, a vacuum of 8E-6 Torr, and an argon (Ar) pressure of 20 sccm. Next, a 10 nm HZO film is subsequently synthesized using plasma-enhanced atomic layer (PE-ALD) deposition, keeping HfO_2_ and ZrO_2_ deposited alternately with the same composition (Hf:Zr = 1:1). The equipment model was PE ALD R200, from Picosun (Espoo, Finland). The growing temperature was 250 °C, and the RF power was 3 kW. An Al_2_O_3_ layer of 2 nm was deposited onto the HZO film. The deposited temperature was 300 °C. The rest of the process conditions were consistent with the HZO deposition process. Finally, the device’s top electrode, 50 nm TiN, was grown by DC magnetron sputtering and patterned by covering a shadow mask. Deposition conditions consisted of a DC power of 500 W, a growth temperature of 300 °C, N_2_ atmosphere, and an argon (Ar) pressure of 20 sccm. The dimensions of each individual top electrode measured 200 μm. The electrical testing of the device was conducted at room temperature with a Keithley 4200 semiconductor analyzer (Keithley, Beaverton, OR, USA).

The following instruments were used in the study of the structural properties of the device in this work. The scanning electron microscope (SEM) (SU8600 produced by Hitachi, Tokyo, Japan) was used to study the cross-sectional morphology and element fraction. X-ray diffraction (XRD) (Smartlab, Tokyo, Japan) was used for the study of the crystal phase of the switching layer. The tapping mode was used during atomic force microscope (AFM) characterization. The instrument model was Bruker’s Dimension Icon (Bruker, Hatfield, PA, USA), in which the probe uses the RTESP series probe.

## 3. Results

**Figure 1:**
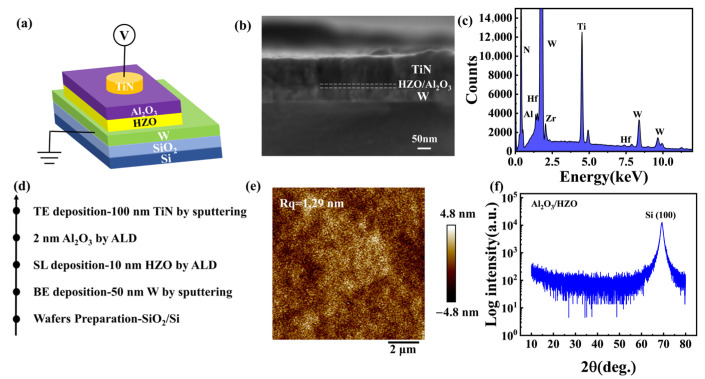
(**a**) The schematic structure of TiN/Al_2_O_3_/HZO/W/Si/SiO_2_ memristor. (**b**) SEM cross-section image of the device. (**c**) EDS face scan spectrum. (**d**) Process flow. (**e**) AFM image of the device. (**f**) XRD of Al_2_O_3_/HZO films deposited on SiO_2_/Si substrate.

Figure 1a shows the schematic of TiN/Al_2_O_3_/HZO/W memristor cells. The SEM cross-section image is shown in Figure 1b, where the thickness of each layer is measured. To probe the element information of the device, EDS face scan spectrum analysis is carried out, as seen in Figure S1. The spectrum in Figure 1c confirmed the atomic and weight fraction of Hf, Zr, Al et al., as detailed in Table S1. Figure 1d shows the process flow of the device, and the predetermined thickness of each film aligns closely with the measurements obtained by SEM. To study the roughness of the resistive layer, the Al_2_O_3_/HZO surface without top electrode was scanned using atomic force microscopy (AFM). The roughness of the Al_2_O_3_/HZO layers measured by AFM is 1.29 nm, as illustrated in Figure 1e. To show the drop of the film more clearly, a line profile was plotted in Figure S2. The film is relatively flat in a randomly selected section. Next, the Al_2_O_3_/HZO layers on Si/SiO_2_ substrate are characterized by XRD from 10 to 80°, as shown in Figure 1f. The absence of further prominent peaks, aside from the Si (100) diffraction peak, suggests that the switching layers are amorphous.

**Figure 2:**
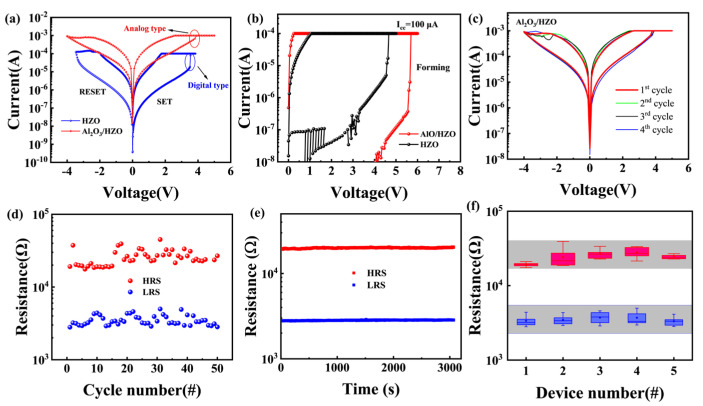
(**a**) Comparison of DC characteristics between HZO-based and Al_2_O_3_/HZO-based memristors. (**b**) Forming process. (**c**) *I*–*V* curves of the Al_2_O_3_/HZO-based memristor at a *I_cc_* of 1 mA. (**d**) The cycle-to-cycle behavior (endurance) of this device. (**e**) Retention characteristics measured over 3000 s. (**f**) Box plots of resistance states for five randomly selected devices.

Figure 2 shows the DC sweep characteristics of the device. The application of a voltage ranging from −5 V to 5 V to the Al_2_O_3_/HZO-based device resulted in bipolar non-volatile resistive switching behavior. In order to compare with the HZO-based memristor, we prepared a device without an Al_2_O_3_ layer and performed electrical tests. As shown in Figure 2a, compared with the binary switching behavior of the HZO-based device, the Al_2_O_3_/HZO-based device exhibited good analog-type switching behavior. The memristor based on Al_2_O_3_/HZO has no significant jump in resistance at 3.5 V.

**Figure 3:**
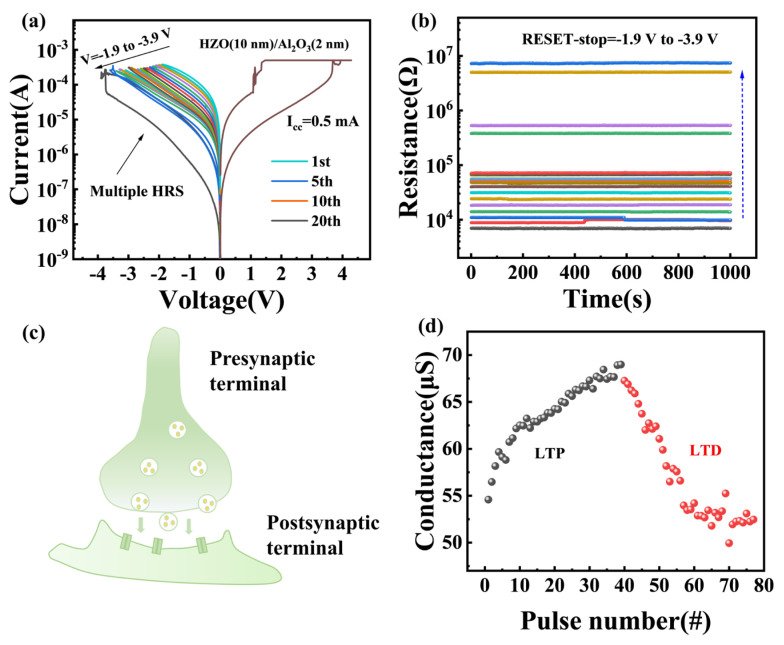
(**a**) Gradual variation of *V_stop_* to get multiple HRS. (**b**) Retention test of 20 resistance states extracted from (**a**). (**c**) The schematic of biologic synapse. (**d**) Long-term potentiation and depression (LTP, LTD) characteristics of the artificial synapse.

This device is in a high-resistance state in the initial state and requires a voltage of around 5 V and a compliance current (*I_cc_*) of 100 μA to transition to a low-resistance state, which is higher than the forming voltage (4 V) of the HZO-based device, as shown in Figure 2b. Subsequent to the forming process, the TiN/Al_2_O_3_/HZO/W memristor is capable of high and low resistance switching (SET and RESET processes) at a positive voltage of 3.8 V (*V_set_*), a negative voltage of −4 V (*V_reset_*), and an *I_cc_* of 1 mA. Figure 2c shows that the device exhibits excellent consistency within 4 cycles. During the RESET process, there is a jump in the resistance value, such as cycle 3. Figure S3 shows around 50 DC scan cycles. When the RESET process was carried out around 50 times, the switching behavior changes significantly. The stability of the RESET process is weaker than that of the SET process, which may be due to the absence of *I_cc_* [29]. To further verify the stability and uniformity of this device, the endurance test was done at *V_read_* of 0.2 V for 50 cycles. As shown in Figure 2d, the device exhibits stable high and low resistance states. Next, the resistance state retention test was performed. Figure 2e illustrates that the retention time for both the high and low resistance states exceeded 3000 s. Device-to-device data was counted, as shown in Figure 2f. There was no obvious jump in the high and low resistance states among the five different devices. The multi-resistance state characteristics are essential for high-density storage applications. Figure 3a shows analog switching by varying *V_stop_* from −1.9 to −3.9 V (0.1 step), generating multiple resistance states. A total of 20 resistance states were extracted and characterized with a retention test, as shown in Figure 3b, which indicates the potential of the device in emulating synaptic functions [30,31,32]. Figure 3c is the schematic of a biological synapse between a pre-synaptic neuron and a post-synaptic neuron. Then a synaptic response test on this device was conducted by an electrical pulse scheme [33,34], with the results shown in Figure 3d. The voltage pulses consist of a pulse width of 20 ms, a pulse amplitude of 4 V, and a total of 40 pulse numbers for the potentiation or depression process.

**Figure 4:**
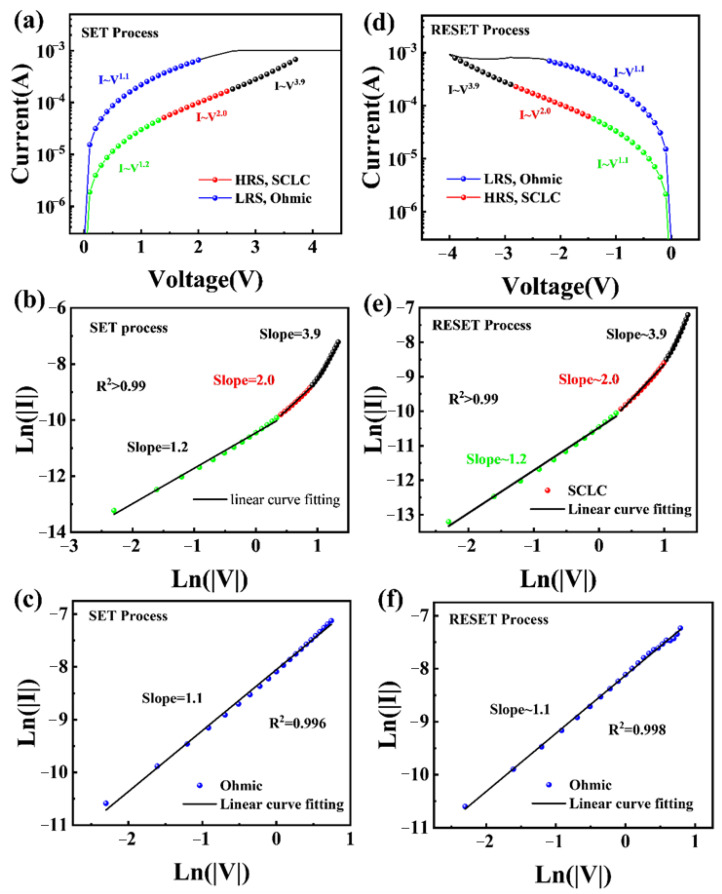
(**a**) Conductive mechanism corresponding to the SET process. (**b**) SCLC conduction fitting curve during SET process. (**c**) Ohmic conduction fitting curve during SET process. (**d**) Conductive mechanism corresponding to the RESET process. (**e**) SCLC conduction fitting curve during RESET process. (**f**) Ohmic conduction mechanism fitting curve during RESET process.

In order to explain the conduction mechanism of the TiN/Al_2_O_3_/HZO/W device, physical models are used to fit the *I*–*V* curve, as shown in Figure 4. In the SET process, the *I*–*V* curve is segmented and fitted in a double log plot, as shown in Figure 4a–c. During the forward voltage scan, as the voltage increases, the slope of the fitting curve transitions from 1.2 to 2.0, as shown in Figure 4b. The color of each curve in the figures corresponds to Figure 4a. This is consistent with the classic space-charge-limited current (SCLC) model [35,36], and the mathematical representation can be expressed as:

(1) $$J=\frac{9}{8}\varepsilon \mu \frac{V^{2}}{d^{3}}$$

where ε is the absolute dielectric constant, V is the voltage, and d is the Boltzmann constant. Then the slope changes to 3.9, which is attributed to the injected charge carriers quickly occupying the shallow charge traps [37]. Upon transitioning from high resistance to low resistance, the slope of the fitting is 1.1, which is consistent with the ohmic contact behavior. In the RESET process, the low resistance state initially follows Ohm’s law, as shown in Figure 4e. When it changes to high resistance, the fitting results are consistent with the SCLC model, as shown in Figure 4f. The color of each curve in the figures corresponds to Figure 4d. The R^2^ values of the above fittings exceed 0.99, proving the credibility of the fitting results.

**Figure 5:**
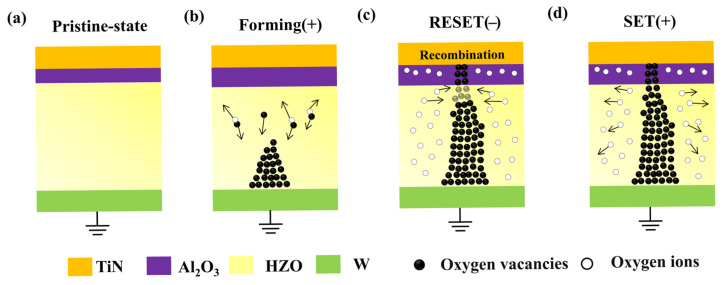
The schematic of switching process in (**a**) Pristine state. (**b**) Forming stage. (**c**) RESET stage. (**d**) SET stage.

Based on the results above, we can conclude that the switching behavior of the TiN/Al_2_O_3_/HZO/W device is dominated by the formation and breakage of the conductive filament in the resistive switching layer. In the absence of an active electrode, the formation of a metallic conductive filament is precluded, resulting in the conductive filament being predominantly constituted of oxygen vacancies [38,39]. In the initial state, the device exhibits a high resistance state, as shown in Figure 5a. The reason for the change of switching behavior from digital to analog can be explained by the difference in the mobility of oxygen vacancies in the bilayer system [40]. In the electroforming process, oxygen vacancies accumulate near the bottom electrode due to the electric field, while oxygen ions move in the opposite direction. The width of the conductive path is reduced from bottom to top due to the higher mobility of the oxygen vacancy in the HZO than in Al_2_O_3_. At this moment, the device transitions from HRS to LRS, as shown in Figure 5b. When a negative voltage is applied, the conductive filament does not completely dissolve in the resistive layer, which explains the reduced switching ratio of the Figure 2a. The oxygen vacancies gradually combine with the oxygen ions, and the conductive filament at the interface of HZO and Al_2_O_3_ gradually disappears, that is, the RESET process, as shown in Figure 5c. Upon reapplication of the forward voltage, the SET process transpires within the device. The conductive filaments do not form suddenly, so they show a gradual switching process, as shown in Figure 5d. Therefore, the TiN/Al_2_O_3_/HZO/W device exhibits analog switching behavior.

Table 1 compares this work with previously reported multilevel resistive switching work.

## 4. Conclusions

This work focuses on the study of the switching characteristics of oxide-based memristors. In order to better simulate the function of neural synapses, the device needs to exhibit analog switching behavior rather than binary type. The TiN/Al_2_O_3_/HZO/W structured memristor proposed in this paper can continuously change from a high resistance state to a low resistance state, avoiding sudden changes. The high and low resistance states of the device have no obvious jumps for more than 3000 s, and have excellent consistency under five different devices. By applying a continuous termination voltage, the device can obtain up to 20 resistance states. In addition, LTP and LTD synaptic behaviors are simulated by applying pulses in opposite directions. The inhibition and enhancement changes are continuous and have good symmetry. Through the I-V curve and model fitting, the switching behavior of the device conforms to the SCLC mechanism. It can be concluded that the high and low resistance state transitions of the device are mainly dominated by the formation and breakage of oxygen vacancy conductive pathways.

## Figures and Tables

**Table 1:** Comparison of this work with previously reported oxide-based work.

Device Structure	Digital/Analog	Switching Mode	Multi-States	Fabrication	Ref.
Ti/HZO/Pt	Digital	Bipolar	No	Sputtering	[41]
Pt/WSe_2_/HZO/TiN	Digital	Bipolar	Yes	ALD	[38]
TiN/TiOx/TiO_2_/TiN	Analog	Bipolar	No	ALD	[22]
Cu/Al_2_O_3_/Si	Digital	Bipolar	No	ALD	[42]
TiN/Al_2_O_3_/HZO/W	Analog	Bipolar	Yes	ALD	This work

## Data Availability

The original contributions presented in this study are included in the article and Supplementary Materials. Further inquiries can be directed to the corresponding authors.

## Supplementary Materials

The following supporting information can be downloaded at: https://www.mdpi.com/article/10.3390/ma18184352/s1, Figure S1: EDS face scan test; Figure S2: The line profile of the AFM image in Figure 1e; Figure S3: 50 DC cycles of Al2O3/HZO based memristor; Table S1: Weight and atomic fraction of all elements determined by EDS.
