# Supplementary material for: Controlling the Digital to Analog and Multilevel Switching in Memristors Based on Zr-Doped HfO2 by Interface Engineering
Source: Materials (Basel). 2025 Sep 17;18(18):4352. doi: 10.3390/ma18184352 (PMC12472119; doi:10.3390/ma18184352)
Supplement: Supplementary file 1 [file materials-18-04352-s001.zip › materials-3801724-supplementary.pdf]

Supporting Information

Controlling the Digital to Analog and Multilevel Switching in Oxide-Based Memristors by Interface Engineering

EDS test:

First, the bottom electrode layer (W) and the switch layer (Al2O3/HZO) are both deposited without patterning. The top electrode layer (TiN) is then formed by the shadow mask. After the device was fabricated, EDS surface scanning is performed on the patterned electrodes of the device to obtain the distribution and fraction of each element.

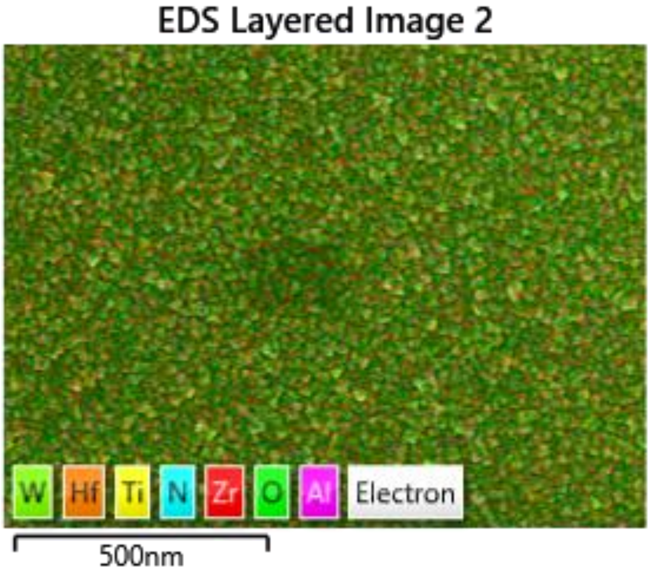

Figure S1. EDS face scan test.

Table S1. Weight and atomic fraction of all elements determined by EDS.

Element	Signal Type	Wt%	Wt% Sigma
N	EDS	28.09	0.34
O	EDS	5.97	0.23
Al	EDS	3.40	0.05
Ti	EDS	16.53	0.15
Zr	EDS	2.49	0.18

Hf	EDS	2.99	0.26
W	EDS	40.54	0.37

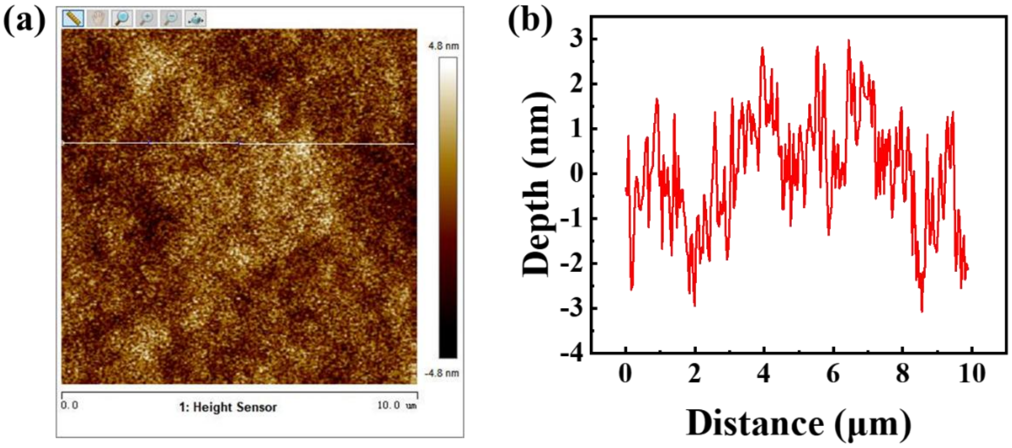

Figure S2. The line profile of the AFM image in Fig. 1e.

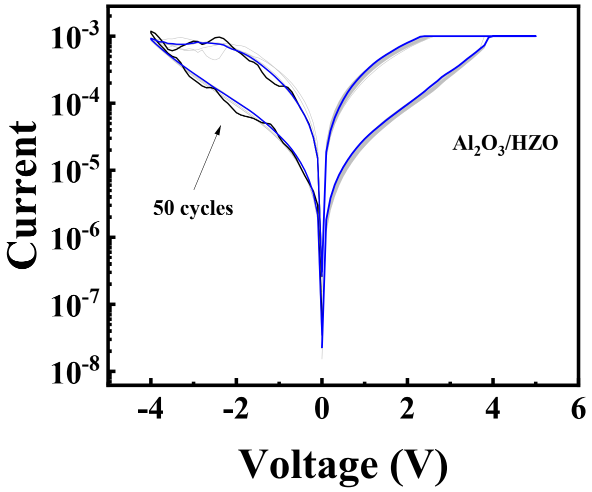

Figure S3. 50 DC cycles of Al_2O_3/HZO based memristor.
